# Basal ganglia engagement during REM sleep movements in Parkinson’s disease

**DOI:** 10.1038/s41531-022-00382-z

**Published:** 2022-09-12

**Authors:** Ajay K. Verma, Sergio Francisco Acosta Lenis, Joshua E. Aman, David Escobar Sanabria, Jing Wang, Amy Pearson, Meghan Hill, Remi Patriat, Lauren E. Schrock, Scott E. Cooper, Michael C. Park, Noam Harel, Michael J. Howell, Colum D. MacKinnon, Jerrold L. Vitek, Luke A. Johnson

**Affiliations:** 1 Department of Neurology, University of Minnesota, Minneapolis, MN USA; 2 Department of Radiology, University of Minnesota, Minneapolis, MN USA; 3 Department of Neurosurgery, University of Minnesota, Minneapolis, MN USA

**Keywords:** Basal ganglia, Parkinson's disease, Neurophysiology

## Abstract

To elucidate the role of the basal ganglia during REM sleep movements in Parkinson’s disease (PD) we recorded pallidal neural activity from four PD patients. Unlike desynchronization commonly observed during wakeful movements, beta oscillations (13–35 Hz) *synchronized* during REM sleep movements; furthermore, high-frequency oscillations (150–350 Hz) synchronized during movement irrespective of sleep-wake states. Our results demonstrate differential engagement of the basal ganglia during REM sleep and awake movements.

## Introduction

The basal ganglia play a critical role in the control of motor function during wakefulness^1,2^. Voluntary movements are preceded by desynchronization (reducing in power) of beta oscillations (13–35 Hz) in the globus pallidus internus (GPi)^3,4^. Excessive spontaneous synchronization of beta oscillations (increasing in power) in the GPi in people with Parkinson’s disease (PD) is thought to contribute to akinesia and bradykinesia since the suppression of beta power by dopamine replacement therapy or deep brain stimulation (DBS) improves motor behavior^4–6^. High-frequency oscillations (HFOs, >100 Hz) in the GPi have been observed to synchronize during wakeful movement in unmedicated PD patients^4,7^. Furthermore, a recent study in PD patients on and off medication, and in naïve and parkinsonian non-human primates suggests that exaggerated HFOs have pathophysiological relevance in PD^4^. Our understanding regarding the modulation of beta oscillations in the basal ganglia during REM sleep movements is limited^8^, however, and HFOs in such context have not been investigated.

In contrast to wakefulness, people with PD can demonstrate improved motor activity during rapid eye movement (REM) sleep^9^. In particular, a high percentage of individuals with PD have REM sleep behavior disorder (RBD), a parasomnia characterized by the loss of muscle atonia and a dramatic increase in motor activity, often with punching and kicking behavior suggestive of dream enactment^10^. People with PD without a clinical diagnosis of RBD may also demonstrate motor activity during REM sleep^9,11^. Currently, little is known about the role of the basal ganglia in the control of movements that occur during REM sleep. Recordings from the subthalamic nucleus have provided initial evidence of marked differences in the dynamics of movement-related oscillations between wake and sleep states^8^. To further explore the role of the basal ganglia in movement control during REM sleep, we recorded local field potentials (LFPs) from four PD patients via externalized directional deep brain stimulation (DBS) leads implanted in the GPi (the principal output nucleus of the basal ganglia). We characterized the dynamics of beta and HFOs in the GPi during REM sleep and wake movements in an effort to improve our understanding of the functional role of the basal ganglia oscillations underlying movement across sleep-wake states.

All subjects were noted to display body movements during REM sleep as identified by video-polysomnography. The number of movements identified during REM sleep, predominant motor phenomena observed, and other demographic information for each subject are reported in Table 1. One subject (#2) had a documented history of sleep dysfunction and displayed complex movements suggestive of dream enactment.

**Table 1:** Demographic data at time of study.

					UPDRS-III			Time and amount (mg) of last Ldopa dose				
Subect	Age/Sex	Disease Dur. (y)	Motor subtype	DBS Side	OFF	ON	Pre Surgery LED (mg)	Night 1 (N1)	Night 2 (N2)	Time in REM (min) N1/N2	No. of REM sleep movements	Predominant REM motor phenomena
1	52/M	6	AR	Right	39	16	750-1250	23:45 (150 mg)	NA	67/NA	33/NA	Simple movements of lower extremeties and body jerks
2	60/F	10	AR	Right	39	17	1200	19:35 (300 mg)	21:30 (300 mg)	45/0	50/0	Bilateral jerk and twitch sequences in the upper and lower extremeties (predominant left hand movements), and head shaking. Apparent dream enactment, complex movements, and vocalization
3	55/M	6	AR	Right	17	12	1160	20:48 (100 mg)	21:30 (145 mg)	95/118	13/12	Twitches and simple movements of foot
4	63/F	7	T	Left	44	19	700	18:03 (300 mg)	13:24 (500 mg)	63/38	21/12	Twitches and simple movements of upper extremeties and simple movements of lower extremeties

*AR* Akinetic-Rigid, *T* Tremor dominant.

**Fig. 1:**
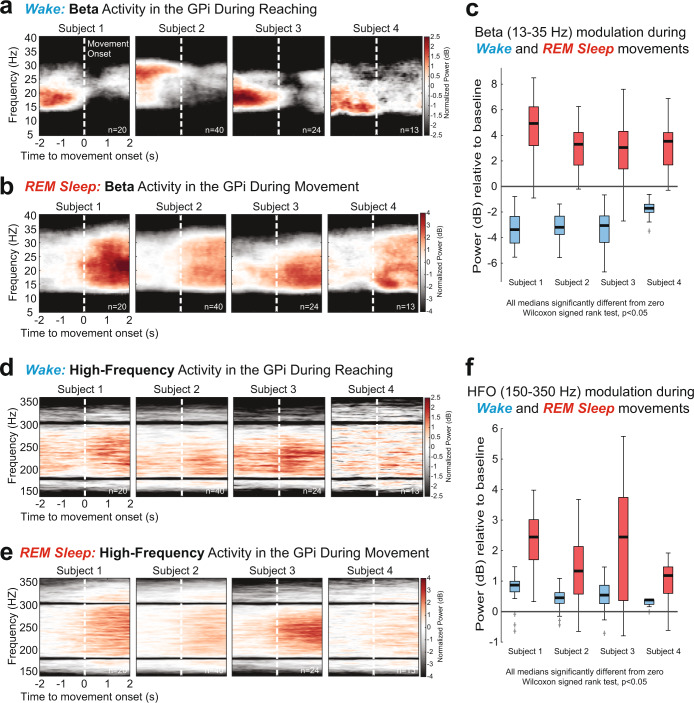
Movement-related beta and high-frequency oscillations recorded from DBS leads in the GPi of PD patients in awake and REM sleep states. **a** Trial-averaged spectrograms aligned to movement onset showing beta (13–35 Hz) desynchronization in the GPi during wakeful volitional movement (reaching task, see Methods). **b** Spectrograms showing beta synchronization in the GPi during REM sleep movements. **c** Distributions of beta band power modulation (relative to pre movement baseline) during wake movements (blue) and REM sleep movements (red). All data distributions were significantly different from zero (Wilcoxon signed rank (WSR) test, *p* < 0.05). **d**, **e** Trial-averaged spectrograms aligned to movement onset show synchronization of high-frequency oscillations (HFO, 150–350 Hz) in the GPi during wakeful and REM sleep movements, respectively. **f** Distributions of HFO band power modulation (relative to pre movement baseline) during wake movements (blue) and REM sleep movements (red). All data distributions were significantly different from zero (WSR test, *p* < 0.05). Boxplot elements: center line, median; box limits, upper and lower quartiles; whiskers, 1.5 × interquartile range; +sign, outliers.

The contact pair on the directional DBS lead exhibiting the highest modulation of beta oscillations for each patient during awake voluntary movements was chosen for analysis and reported in Fig. 1. Additional details regarding beta and HFO modulation associated with REM sleep and awake movements for other contact pairs on the directional DBS lead, and lead location in the GPi, are summarized in Supplementary Fig. 1.

Trial-averaged spectrograms depicting oscillatory power changes in the beta band (13–35 Hz) during awake voluntary movements (reaching task, see Methods for details) and REM sleep movements are shown in Fig. 1a, b, respectively. In the awake condition, beta oscillations significantly desynchronized during movement execution in all subjects compared to a pre-movement period (Wilcoxon signed rank (WSR) test, *p* < 0.05, Fig. 1c, blue boxplots). Conversely, all participants exhibited strong synchronization of beta oscillations during REM sleep movements compared to baseline (WSR test, *p* < 0.05 Fig. 1c, red boxplots). In contrast to the beta band, which showed opposite polarity of modulation during wake and REM sleep movements, HFOs (150–350 Hz) showed movement-related synchronization during both wake and REM sleep (Fig. 1d, e).

The primary finding of this study was that, in people with PD, movement-related beta oscillations in the GPi synchronized during REM sleep but desynchronized during wakefulness. In contrast, HFOs in the GPi synchronized during movement regardless of sleep-wake state. Taken together, our results demonstrate that GPi is engaged during REM sleep movements and question the hypothesis that the basal ganglia is not involved in the generation of movements during REM sleep in people with PD^9^.

Previous studies have reported improved motor behavior during REM sleep in PD patients with RBD^9,12^. Furthermore, motor events generated during REM sleep in PD patients with RBD and idiopathic RBD patients were observed to be qualitatively similar^13^. These observations led to a hypothesis that the dopamine deficient basal ganglia may not be involved in the movements generated during REM sleep in people with PD^9,12^. Potential electrophysiological support for this idea comes from a study in four PD patients with RBD showing movement-related synchronization of the subthalamic nucleus (STN) beta oscillations during REM sleep^8^. Since STN beta oscillations in the basal ganglia desynchronize during wakeful movements, which is thought to be permissive to the movement^3,8,14^, the authors interpreted their data as supportive of the hypothesis that pathological basal ganglia signaling is bypassed and the movements during REM sleep are mediated by pathways alternate to the cortico-basal ganglia network in people with PD.

We observed significant synchronization of beta oscillations in the GPi during REM sleep movements in all four subjects, which could be viewed as providing additional support for the hypothesis that basal ganglia is not involved in the generation of movements during REM sleep. Our findings of movement-related HFO synchronization in the GPi during both wake and REM sleep, however, complicate this interpretation. Elevated HFO power has been shown to be a characteristic feature of movements during wakefulness in PD^4,7,15^. If movements during REM sleep are not mediated by the basal ganglia, then the polarity of movement-related modulation of HFOs during REM sleep might be expected to be opposite to wakefulness as was observed with beta oscillations. However, the polarity of movement-related modulation of HFOs in the GPi during REM sleep was similar to that during wakefulness i.e., synchronizing during movement irrespective of sleep-wake states. We interpret these findings as demonstrating that the basal ganglia is likely engaged during REM sleep movements, but its role in movement control differs greatly during wakefulness and REM sleep.

Furthermore, we observed that the polarity of modulation in beta and HFOs between PD patients with (*n* = 1) and without (*n* = 3) RBD were qualitatively similar. This leads us to speculate that synchronization of beta oscillations and HFOs in response to movements generated during REM sleep are characteristic of REM sleep in PD and may not be unique to PD patients diagnosed with RBD. A future study with a higher sample size of PD patients with and without RBD will be required to confirm this observation.

Despite our understanding of skeletal muscle atonia during REM sleep, small movements during REM sleep is possible in healthy individuals^16,17^. Whether the movement-related changes in beta oscillations and HFOs we observed during REM sleep are specific to PD or more generally applicable remains unclear. Identification of these events with concomitant recordings across the basal ganglia-cortical motor network can shed further light on the neural mechanisms of movement control during REM sleep and enhance our understanding of physiological and pathological movements generated during sleep. While it is not feasible to perform such electrophysiological recordings in healthy controls, data collection may be feasible from patients with non-PD neurological disorders such as dystonia and Tourette syndrome, where the STN and GPi are common DBS targets^5,18–20^, to understand if movement-related dynamics of beta oscillations and HFOs during REM sleep is unique to PD.

In summary, we showed that, contrary to the stereotypical pattern of movement-related beta desynchronization in the GPi during voluntary wakeful movements, beta oscillations in the GPi are synchronized during REM sleep movements. Furthermore, HFOs are synchronized during movement irrespective of sleep-wake state. Together, our results demonstrate differential engagement of pallidum during wake and REM sleep movements and these findings may inform the development of DBS approaches tailored to suppress excessive movements generated during REM sleep in people with PD.

## Methods

This study was approved by the University of Minnesota Institutional Review Board (#1701M04144) and informed consent was obtained according to the Declaration of Helsinki. Four participants (two female) with idiopathic PD consented to externalization of their DBS lead. All subjects were implanted unilaterally with a directional DBS lead targeting the GPi. The demographic information for each subject are reported in Table 1.

### Data collection

#### Surgical Procedure

Details of the surgical procedures for GPi DBS implantation and lead externalization are described in detail in previous publications^4,21^. Briefly, subjects underwent standard 3 T MRI (all subjects) and a high resolution 7 T MRI (excluding Subject 3) for direct targeting and postoperative lead localization^22,23^. Intraoperative electrophysiological mapping techniques were used to identify the sensorimotor region of GPi for implantation^24^. In all subjects a directional “1-3-3-1” electrode was used (Subjects 1,2,4: Abbott Infinity model 6172, illustrated in Supplementary Fig. 1a; Subject 3: Boston Scientific Vercise Cartesia model DB-2202-45; all leads had 1.5 mm contact height with 0.5 mm vertical spacing). After implantation, the lead was connected to an extension wire which was tunneled to a subcutaneous pocket in the chest and then connected to another extension wire which was externalized at the abdomen (“percutaneous extension”)^21^. Externalized components were secured and protected with a water-proof barrier dressing and subjects were discharged to home to recover. Externalization recordings occurred 4–8 days later, allowing some time for reduction of microlesion effects that can occur following lead placement^25,26^. After the study, subjects returned to the hospital for removal of the percutaneous or externalized extension wire and placement of the implantable pulse generator (IPG). A movement disorders clinician performed DBS programming approximately 4-6 weeks after IPG placement per standard clinical care.

#### Sleep recording and staging

LFP activity from the DBS lead and scalp electroencephalography (EEG, 10–20 montage, modified as needed to accommodate scalp incision) were collected over the course of two days while residing in the University of Minnesota Health Clinical Research Unit. Signals were recorded on an Xltek NeuroWorks Workstation (Quantum amplifier, 4096 Hz sampling rate, Natus) to enable post-hoc video-polysomnography (v-PSG) and analysis of time-synchronized pallidal oscillatory activity. v-PSG data (including EEG, electrooculogram (EOG), electromyogram (EMG), video) were imported into Natus SleepWorks software. Sleep staging was performed according to the American Academy of Sleep Medicine (AASM) Manual for the Scoring of Sleep and Associated Events Version 2.6 by a registered polysomnographic technician (A.P.) using the standard EEG, EOG, and chin EMG montage^27^. Sleep stages (Wake, NREM 1 (N1), NREM 2 (N2), NREM 3 (N3), and REM) were first determined in standard 30-s epochs (consistent with AASM scoring guidelines). Subsequently, 10-s mini-epochs were subdivided to better identify brief sleep phenomena including K complexes, sleep spindles, slow-wave activity, phasic REM, and tonic REM. Neural data and sleep stages and sleep phenomena annotations were then exported in EDF format for subsequent analysis.

#### REM sleep movement and reaching task

v-PSG recordings were reviewed by experienced sleep specialists (M.H. and A.P.). Movements occurring during REM sleep epochs were visually identified and characterized (twitches, periodic limb movements, complex dream enactment, limb adjustment, or body position change) and timestamps of movement onset were saved for further analysis of event-related potentials. Predominant motor phenomena for each subject are described in Table 1. As a comparison condition, GPi LFP data collected during a daytime motor task were also analyzed. LFP data were collected while patients performed a simple touchscreen reaching task using the arm contralateral to the implanted DBS lead. Trials began with the hand on a digitized home button located 45 cm from a touchscreen monitor. After a randomized variable 3–4 s delay following the start of a trial, a 1.27 cm hollow circle (target) appeared on the center of the touchscreen along with a 5 cm square box directly to the left of the circle (10 cm). The appearance of the circle and square was the patient’s “go cue”. Subjects were instructed to touch and drag the circle into the square box as quickly and accurately as possible and then return to the home button. The number of reach trials included in the analysis were matched to the number of REM movements detected in each patient and are reported in the figures (see Data analysis and statistics section below for additional details). Wake task-related data were included in previous publications but analyzed differently in the present study^4,21^.

### Data analysis and statistics

All analyses were performed using customized scripts in MATLAB (MathWorks). LFP activity was extracted via bipolar montage (i.e. signal subtraction) of vertically adjacent DBS contacts within the GPi (see Supplementary Fig. 1a); the segmented contacts of the 1-3-3-1 directional lead were used in this study, resulting in three bipolar pairs that were analyzed.

A multimodal exploration of video, EEG (including EOG and EMG), and LFP signals (in time and frequency domain) was done to identify artifact-free movement events during REM. Timestamps of movement initiation during the reaching task were similarly identified. In all subjects, the number of reach trials (*n* = 50) surpassed the number of identified REM sleep movements. The same number of artifact-free samples were collected in chronological order from the data associated with the reaching task (i.e. if 40 REM sleep movements were detected, the first 40 reach movements were analyzed).

LFP signals were filtered between 13 and 35 Hz for the beta oscillations, and between 150 and 350 Hz for the HFOs. Perievent spectrograms aligned to movement onset were computed via the multi-taper method and Chronux toolbox using a moving window of 1 s, 10 ms steps, with three tapers resulting in a frequency resolution of 0.5 Hz^28^. For each subject and frequency band (i.e. beta, HFO) the spectrogram was normalized to the total power in the target frequency band during the 1st s of baseline period and reported in units of dB relative to the baseline^8,29^. To quantify movement-related changes in the normalized spectrogram, for each trial the mean band power in a pre-movement period 2 s before movement onset (AvgPower_Pre-move_) and in a movement period 2-s duration beginning with movement onset (AvgPower_Move_) were calculated. Boxplots display distributions of normalized trial-by-trial movement-related band power modulation (AvgPower_Move –_ AvgPower_Pre-move_). Non-parametric statistical tests were performed because of the relatively small sample size and the non-normality of the data. To determine whether a subject’s median movement-related modulation was significantly different from zero, reflecting significant synchronization (positive value) or desynchronization (negative value) in the frequency band of interest, the Wilcoxon signed-rank test was performed (*p* < 0.05). The segment pair in each subject with the greatest movement-related modulation in the beta band is presented in the main results section and Fig. 1. Movement-related modulation from all three segment pairs for each subject is presented in Supplementary Fig. 1. To test whether there is some spatial specificity between recording directions in the GPi, the sleep and wake movement power modulations from the three directions were compared using the Kruskal–Wallis test (*p* < 0.05), testing the null hypothesis that all three distributions come from the same distribution, with post-hoc pairwise comparisons between directions made with Bonferroni correction for multiple comparisons.

### Determining DBS lead orientation

DBS lead locations in the GPi were estimated based on information obtained during intraoperative electrophysiological mapping as well as co-registered preoperative MRI and postoperative CT scans (see ref. ^23^ for details). The orientation of the DBS lead and relative direction of individual segments for each patient were derived from the fiducial marker on the lead, in combination with the unique artifact characteristics of the segments, using a modified version of the DiODe algorithm^30^. The original DiODe algorithm was designed and validated for the Boston Scientific Cartesia electrodes; since most of our patients were implanted with the Abbott Infinity electrodes, the MATLAB code was modified to be compatible with the new electrode characteristics (e.g., smaller marker and slight changes in the intensity profiles of the segments artifacts) in collaboration with Dr. Dembek, the lead author of the DiODe algorithm (personal communications, R.P.). Additional details can be found in ref. ^4^.

## Supplementary information

Supplementary Figure 1

## Data Availability

The data that support the findings of this study are available from the corresponding author upon reasonable request.

## Code availability

The code associated with the findings of this study are available from the corresponding author upon reasonable request.

## References

[1] Brittain JS, Brown P (2014). Oscillations and the basal ganglia: motor control and beyond. Neuroimage.

[2] Groenewegen HJ (2003). The basal ganglia and motor control. Neural Plast..

[3] Eisinger RS (2020). Parkinsonian beta dynamics during rest and movement in the dorsal pallidum and subthalamic nucleus. J. Neurosci..

[4] Johnson, L. A. et al. High‐frequency oscillations in the pallidum: a pathophysiological biomarker in Parkinson’s Disease? *Mov. Disord*. **36**, 1332–1341 (2021). 10.1002/mds.28566 PMC8422942 33847406

[5] Wang DD (2018). Pallidal deep-brain stimulation disrupts pallidal beta oscillations and coherence with primary motor cortex in Parkinson’s disease. J. Neurosci..

[6] Cagle, J.N. et al. Suppression and rebound of pallidal beta power: observation using a chronic sensing DBS device. *Front. Hum. Neurosci*. **15**, 749567 (2021). 10.3389/fnhum.2021.749567 PMC8458625 34566612

[7] Tsiokos C, Hu X, Pouratian N (2013). 200–300 Hz movement modulated oscillations in the internal globus pallidus of patients with Parkinson’s Disease. Neurobiol. Dis..

[8] Hackius M, Werth E, Sürücü O, Baumann CR, Imbach LL (2016). Electrophysiological evidence for alternative motor networks in REM sleep behavior disorder. J. Neurosci..

[9] De Cock VC (2007). Restoration of normal motor control in Parkinson’s disease during REM sleep. Brain.

[10] Howell MJ, Schenck CH (2015). Rapid eye movement sleep behavior disorder and neurodegenerative disease. JAMA Neurol..

[11] Sringean, J. et al. Rapid eye movement sleep behavior disorder and rapid eye movement sleep without atonia are more frequent in advanced versus early Parkinson’s disease. *SLEEPJ* **44**, zsab067 (2021). 10.1093/sleep/zsab067 33720377

[12] Cochen De Cock V (2011). The improvement of movement and speech during rapid eye movement sleep behaviour disorder in multiple system atrophy. Brain.

[13] Bugalho P (2017). Characterization of motor events in REM sleep behavior disorder. J. Neural Transm..

[14] Kühn AA (2004). Event‐related beta desynchronization in human subthalamic nucleus correlates with motor performance. Brain.

[15] AuYong N, Malekmohammadi M, Ricks-Oddie J, Pouratian N (2018). Movement-modulation of local power and phase amplitude coupling in bilateral globus pallidus interna in Parkinson Disease. Front. Hum. Neurosci..

[16] Stefani A (2015). A prospective video-polysomnographic analysis of movements during physiological. Sleep. 100 Healthy Sleepers. Sleep..

[17] Frauscher B (2014). Motor events during healthy sleep: a quantitative polysomnographic study. Sleep.

[18] Deng Z (2018). Subthalamic deep brain stimulation in patients with primary dystonia: a ten-year follow-up study. Parkinsonism Relat. Disord..

[19] Tsuboi T, Jabarkheel Z, Foote KD, Okun MS, Wagle Shukla A (2019). Importance of the initial response to GPi deep brain stimulation in dystonia: a nine year quality of life study. Parkinsonism Relat. Disord..

[20] Xu W (2020). Deep brain stimulation for Tourette’s syndrome. Transl. Neurodegener..

[21] Aman JE (2020). Directional deep brain stimulation leads reveal spatially distinct oscillatory activity in the globus pallidus internus of Parkinson’s disease patients. Neurobiol. Dis..

[22] Duchin Y (2018). Patient-specific anatomical model for deep brain stimulation based on 7 Tesla MRI. PLOS One.

[23] Patriat, R. et al. Individualized tractography-based parcellation of the globus pallidus pars interna using 7T MRI in movement disorder patients prior to DBS surgery. *Neuroimage* **178**, 198–209 (218). 10.1016/j.neuroimage.2018.05.048 PMC6046264 29787868

[24] Vitek JL (1998). Microelectrode-guided pallidotomy: technical approach and its application in medically intractable Parkinson’s disease. J. Neurosurg..

[25] Koop, M. M., Andrzejewski, A., Hill, B. C., Heit, G., & Bronte‐Stewart, H. M. Improvement in a quantitative measure of bradykinesia after microelectrode recording in patients with Parkinson’s disease during deep brain stimulation surgery. *Mov. Disord*. **21**, 673–678 (2006). 10.1002/mds.20796 16440333

[26] Vitek JL (2020). Subthalamic nucleus deep brain stimulation with a multiple independent constant current-controlled device in Parkinson’s disease (INTREPID): a multicentre, double-blind, randomised, sham-controlled study. Lancet Neurol..

[27] Berry, R .B., Brooks, R., & Gamaldo, C. E. *The AASM manual for the scoring of sleep and associated events: rules, terminology and technical specifications, version 2.6. 0* (American Academy of Sleep Medicine, 2020).

[28] Bokil H, Andrews P, Kulkarni JE, Mehta S, Mitra PP (2010). Chronux: a platform for analyzing neural signals. J. Neurosci. Methods.

[29] Goddard, C. A., Sridharan, D., Huguenard, J. R., & Knudsen, E. I. Gamma oscillations are generated locally in an attention-related midbrain network. *Neuron* **73**, 567–580 (2012). 10.1016/j.neuron.2011.11.028 PMC3291715 22325207

[30] Hellerbach A (2018). DiODe: directional orientation detection of segmented deep brain stimulation leads: a sequential algorithm based on CT imaging. Stereotact. Funct. Neurosurg..

