# Supplementary material for: Basal ganglia engagement during REM sleep movements in Parkinson’s disease
Source: NPJ Parkinsons Dis. 2022 Sep 12;8:116. doi: 10.1038/s41531-022-00382-z (PMC9468156; doi:10.1038/s41531-022-00382-z)
Supplement: Supplementary file 1 — Supplementary Figure 1 [file 41531_2022_382_MOESM1_ESM.pdf]

Title:

Basal Ganglia Engagement during REM Sleep Movements in Parkinson's Disease

Running Head:

GPi Activity during REM Sleep and Awake Movements

Author list:

Ajay K. Verma[1]*, Sergio Francisco Acosta Lenis[1]*, Joshua E. Aman[1], David Escobar Sanabria[1], Jing Wang[1], Amy Pearson[1], Meghan Hill[1], Remi Patriat[2], Lauren E. Schrock[1], Scott E. Cooper[1], Michael C. Park[1,3], Noam Harel[2,3], Michael J. Howell[1], Colum D. MacKinnon[1], Jerrold L. Vitek[1], Luke A. Johnson[1†]

*These authors contributed equally

[1] Department of Neurology, University of Minnesota, Minneapolis, Minnesota, USA

[2] Department of Radiology, University of Minnesota, Minneapolis, Minnesota, USA

[3] Department of Neurosurgery, University of Minnesota, Minneapolis, Minnesota, USA

†Corresponding Author:

Luke A. Johnson, PhD

Department of Neurology, University of Minnesota

Lions Research Building 410

2001 6th St SE

Minneapolis, MN 55455

Email: joh03032@umn.edu

Minneapolis, MN 55455

+1 314-479-6700

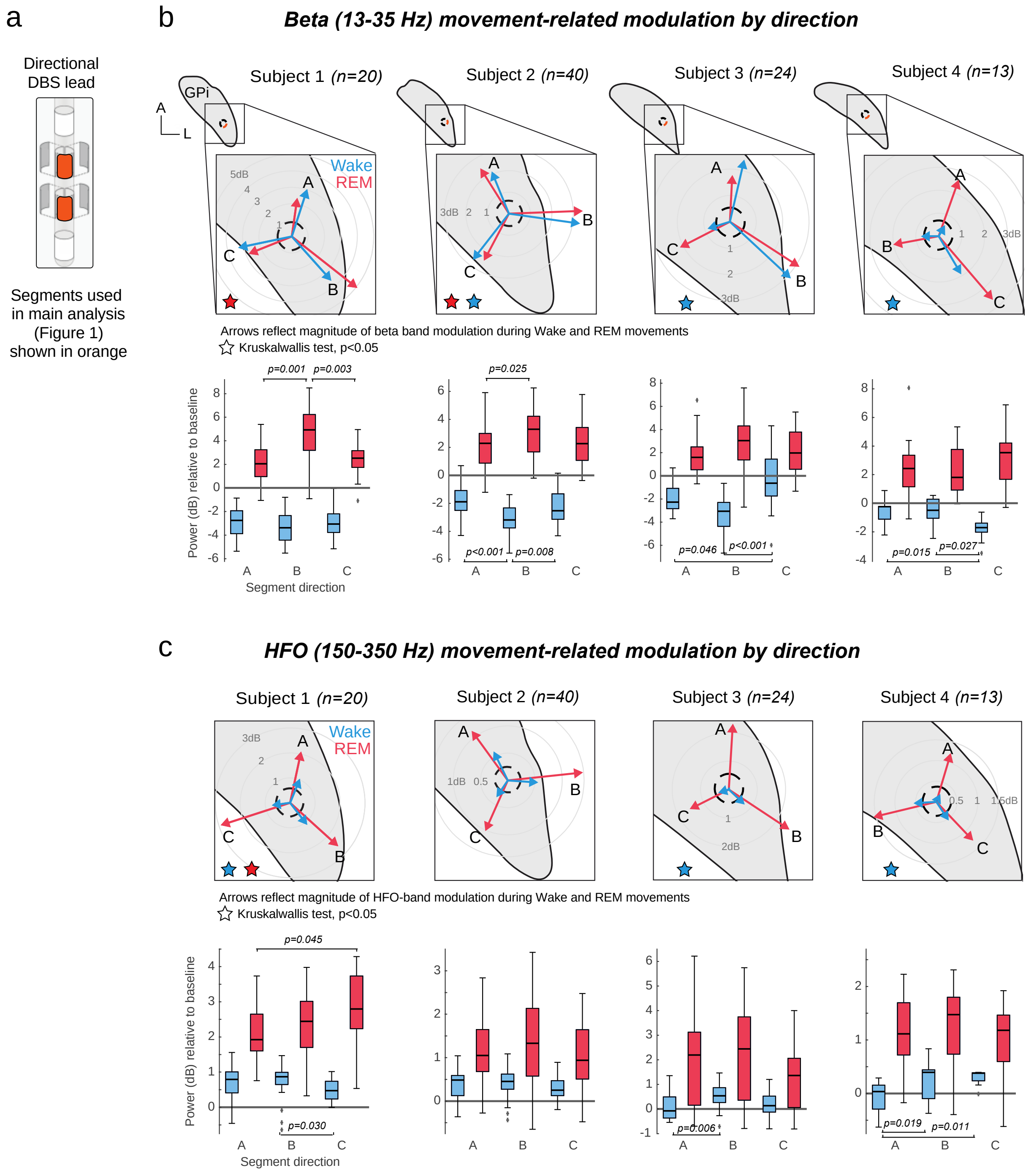

Supplementary Figure 1. GPi DBS recording locations and movement-related beta and high-frequency power modulation by direction.

(a) Schematic of an Abbott directional “1–3–3–1” lead, illustrating in orange that bipolar paired recordings from vertically adjacent segments were used in this study. **(b)** DBS lead implant

locations for each patient, estimated from preoperative MRI and postoperative CT scans. Axial reconstructions are shown, with recording segment used in primary analysis (**Figure 1**, Results section) shown in orange, chosen based on the segment direction with largest movement-related modulation of beta band activity (see Methods). Recordings were made in right GPi in patients 1-3 and in left GPi in patient 4; left GPi images were mirrored for visualization. Subject 3 was implanted with a Boston Scientific directional lead (see Methods) which has alternative contact labeling compared to Abbott, however for visualization in this figure, directions were assigned A, B and C labels. Distributions (lower panels) and median magnitude (upper panels) of beta band modulation in each direction are presented. To determine statistically significant directionality of oscillatory activity, the Kruskal-Wallis test was performed separately on wake and REM sleep datasets to determine if distributions in the three directions come from the same distribution ($p<0.05$), followed by pairwise tests correcting for multiple comparisons. Analogous plots but for high-frequency band power modulations are shown in **(c)**. Boxplot elements: center line, median; box limits, upper and lower quartiles; whiskers, 1.5×interquartile range; +sign, outliers.
